# Respiratory Motion Reduction in PET/CT Using Abdominal Compression for Lung Cancer Patients

**DOI:** 10.1371/journal.pone.0098033

**Published:** 2014-05-16

**Authors:** Tzung-Chi Huang, Yao-Ching Wang, Yu-Rou Chiou, Chia-Hung Kao

**Affiliations:** 1 Department of Biomedical Imaging and Radiological Science, China Medical University, Taichung City, Taiwan; 2 Department of Biomedical Informatics, Asia University, Taichung City, Taiwan; 3 Department of Radiation Oncology, China Medical University Hospital, Taichung City, Taiwan; 4 Department of Nuclear Medicine, China Medical University Hospital, Taichung City, Taiwan; Wayne State University, United States of America

## Abstract

**Purpose:**

Respiratory motion causes substantial artifacts in reconstructed PET images when using helical CT as the attenuation map in PET/CT imaging. In this study, we aimed to reduce the respiratory artifacts in PET/CT images of patients with lung tumors using an abdominal compression device.

**Methods:**

Twelve patients with lung cancer located in the middle or lower lobe of the lung were recruited. The patients were injected with 370 MBq of ^18^F-FDG. During PET, the patients assumed two bed positions for 1.5 min/bed. After conducting free-breathing imaging, we obtained images of the patients with abdominal compression by applying the same setup used in the free-breathing scan. The differences in the standardized uptake value (SUV)_max_, SUV_mean_, tumor volume, and the centroid of the tumors between PET and various CT schemes were measured.

**Results:**

The SUV_max_ and SUV_mean_ derived from PET/CT imaging using an abdominal compression device increased for all the lesions, compared with those obtained using the conventional approach. The percentage increases were 18.1% ±14% and 17% ±16.8% for SUV_max_ and SUV_mean_, respectively. PET/CT imaging combined with abdominal compression generally reduced the tumor mismatch between CT and the corresponding attenuation corrected PET images, with an average decrease of 1.9±1.7 mm over all the cases.

**Conclusions:**

PET/CT imaging combined with abdominal compression reduces respiratory artifacts and PET/CT misregistration, and enhances quantitative SUV in tumor. Abdominal compression is easy to set up and is an effective method used in PET/CT imaging for clinical oncology, especially in the thoracic region.

## Introduction

Respiratory motion causes image artifacts in PET/CT images and misalignment between PET and CT. In PET imaging, respiratory motion may give cause image blurring, degradation in the image contrast, and an overestimation of the lesion volume. In CT, respiratory motion may distort the tumor shape and volume [1]. In addition, when using CT images to correct for attenuation in PET data, the mismatch between PET and CT images caused by respiration may result in errors in localizing the tumor in PET, leading to an inaccurate standardized uptake value (SUV) because of the large difference in the acquisition time of CT and PET. An overestimation of the volume and underestimation of the SUV of a lung lesion caused by respiratory motion were reported by Nehmeh et al and Erdi et al [2]–[3]. Liu et al reported the increased uncertainty of the SUV for lung tumors when attenuation correction (AC) was performed using misaligned PET/CT [4]. Huang et al demonstrated that increased tumor motion is closely associated with the SUV maximum (SUV_max_) decrease in patients with lung cancer [5]. These artifacts and the misalignment could cause potential misdiagnoses when combined with the PET/CT imaging modality for lung cancer diagnosis [6].

Several techniques have been investigated to correct the PET/CT misalignments and reduce artifacts to improve the quantitative accuracy. The respiratory gating of PET and CT, in which the collected data were binned into certain respiratory phases, was used to reduce the motion artifacts and SUV errors [7]–[8]. The results of applying 4-dimensional (4D) PET/CT using 4D-CT data with the gated PET images indicated improved lesion registration and appropriate internal tumor volumes [1], [9]. However, the long acquisition and processing time required to conduct the examination was inevitable. The deep-inspiration breath-hold technique has been proposed to improve the inaccurate quantification of both SUV_max_ and metabolic volume, but this method is not practical for all patients because it requires patient compliance and may not be feasible for patients with limited pulmonary function [6], [10]. Cine average CT (CACT) was proposed for AC in PET and the images exhibited considerably less misalignments and artifacts compared with those obtained using conventional helical CT (HCT)-based AC [11]. The main problem of CACT is that it requires the administration of a relatively high radiation dose. Recently, the interpolated average CT used for PET/CT AC corrected the PET/CT misregistration and enhanced lesion quantitation accompanied by radiation deduction. However, the complicated postimaging process is still a concern regarding the use of these techniques in clinical practice [12]–[14].

Abdominal compression is commonly used for reducing thoracic tumor motion during treatment delivery in radiation oncology. The use of abdominal compression for lung radiation treatments efficiently reduces motion amplitude for lesions close to the diaphragm [15]–[16]. In this study, we demonstrated respiratory motion correction in PET/CT by using an abdominal compression device, and investigated the potential improvement of the results compared with those produced using conventional CT (HCT) on patients with lung cancer.

## Materials and Methods

### Patient Population

The current study was conducted from August 2013 to October 2013. Twelve patients (5 male, 7 female; average age, 60 years; age range, 43–77 years) with a diagnosis of lung cancer confirmed by a physician at China Medical University Hospital were recruited. The lung lesions had a size ranging from 3 to 44 cm. All the patients who were selected had a tumor in the middle or lower lobe of the lung, which are regions in which respiratory motion clearly occurs. A summary of the clinical characteristics of the patients is shown in Table 1. Written informed consent was obtained from all the patients. All the data collection and analyses performed in this study were approved by the Institutional Review Board of China Medical University Hospital.

**Table 1:** Clinical patient characteristics.

Patient no.	Sex	Age(yr)	Lesion location	Lesion volume(cm^3^)
1	M	62	Right lower lobe	8.46
2	F	50	Left lower lobe	17.9
3	M	61	Left lower lobe	6.41
4	M	61	Right lower lobe	17.65
5	F	52	Right lower lobe	4.42
6	F	55	Left lower lobe	41.90
7	F	57	Right middle lobe	3.37
8	F	77	Left upper lobe	6.20
9	M	74	Right lower lobe	44.58
10	F	43	Left lower lobe	16.49
11	M	72	Right lower lobe	6.06
12	F	64	Left lower lobe	40.59

### Imaging Acquisition Protocol

The patients were all injected with 370 MBq of ^18^F-FDG. During the uptake phase that lasted for approximately 40 minutes, the patients remained in a still position. The first whole-body image was obtained when the patients were in a supine position and the acquisition time per bed position was 1.5 min. Free-breathing whole-body CT was conducted at 120 kV in helical mode with a smart mA (range 30–210 mA), 1.75∶1 pitch, and 0.5-s gantry rotation. For the thoracic PET, the patients assumed two bed positions with 1.5 min/bed. After performing free-breathing imaging (<5 min), we obtained images of the patients with abdominal compression by using the same setup as that used in the free-breathing scan.

**Figure 1:**
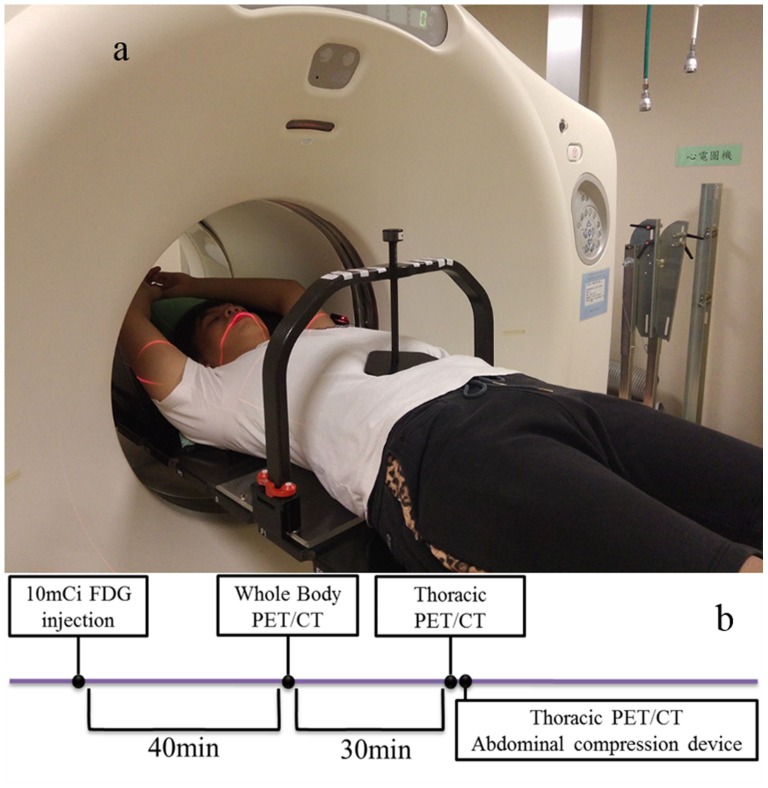
Patient setup with the abdominal compression device (a) located in the upper abdomen region to limit the amount of respiration. (b) The data acquisition protocol for the PET/CT imaging of patients with abdominal compression immediately after conducting free-breathing imaging.

All the scans were acquired using a GE PET/CT-16 slice and a Discovery STE (GE Medical System, Milwaukee, Wisconsin USA) combined with an abdominal compression device (BodyFix Diaphragm Control, Elekta) in 3-dimensional mode with transaxial field-of-views (FOVs) of 70 and 50 cm for PET and CT, respectively. The imaging protocol and the patient setup including the abdominal compression device are shown in Figures 1a and 1b.

We used the same clinical reconstruction parameters for both the free-breathing PET and abdominal-compression PET images. The PET_FB_ and PET_ab_ images were reconstructed using iterative algorithms (Fourier rebinning and attenuation-weighted ordered-subset expectation maximization, two iterations, 20 subsets, and a 6-mm Gaussian filter) and AC using HCT and abdominal-compression CT (CT_ab_), respectively. The data were reconstructed using a 128×128 matrix and a 3-mm-thick slice. All the PET and CT images were transferred to a GE workstation from which fusion PET/CT images were constructed.

### Lesion Analysis

In the 3-dimensional (3D) PET/CT images, a 3D volume-of-interest (VOI) was manually drawn by an experienced physician for each lesion in the PET images [17]. The maximal value and the mean SUV value in the VOI were defined as SUV_max_ and SUV_mean_, respectively. The corresponding delineation of the VOI in the CT images was performed by a radiation oncologist. SUV_max_ was obtained for all lesions shown in the PET_FB_ and PET_ab_ images. The values of the SUV_max_, SUV_mean_, and VOI were compared. The continuous variables were expressed as the mean ± the standard deviation (SD). Statistical analyses were conducted using the unpaired Student’s *t* test and paired *t* test. A *P* value of <0.05 was considered to be statistically significant. In addition, the coordinates of the centroid of the lesion in the PET_FB_, CT and PET_ab_, and CT_ab_ images were determined based on the chosen VOIs. The distances *d* between the tumor centroid in the PET image and the associated CT image were then measured.

## Results

The SUV_max_ and SUV_mean_ for all the tumors are summarized in Table 2. The PET_ab_ image generally showed increased SUV_max_ and SUV_mean_ for all the lesions compared with those shown in the PET_FB_ image. The percentage increase (%diff) was 18.1% ±14% and 17% ±16.8% for SUV_max_ and SUV_mean_, respectively. The percentage difference of tumor volume in PET was in the range of 0.1% to 41%. PET/CT imaging combined with abdominal compression generally reduced tumor mismatch *d* between the CT image and the corresponding attenuation corrected PET images, as shown in Table 2, with an average decrease of 1.9±1.7 mm across all the tumors.

**Table 2:** Summary of the quantitative results obtained using the conventional and abdominal compression methods.

Patient #	SUV_max_			SUV_mean_			Tumor Volume (cm^3^)			d(mm)		
	PET_HCT_	PET_ab_	%diff	PET_HCT_	PET_ab_	%diff	PET_HCT_	PETab	%diff	HCT/PET_HCT_	CT_ab_/PET^ab^	Diff.(mm)
1	3.3	3.9	18	1.8	2.3	28	8.5	5.6	34	5.7	3.7	2.0
2	12.8	14.5	13	7.7	8.7	13	17.9	16.9	6	5.2	4.2	1.0
3	6.0	6.6	10	3.6	3.7	0.1	6.415	3.8	41	5.2	3.6	1.6
4	6.3	6.7	6	3.4	3.9	15	17.7	12.9	27	7.0	6.0	1
5	3.5	4.6	31	2.0	2.3	15	4.4	2.6	41	3.7	3.4	0.3
6	11.2	12.0	7	6.6	6.8	3	41.9	37.4	11	4.9	3.7	1.2
7	6.0	8.0	33	3.5	4.2	20	3.4	2.5	26	3.4	3.2	0.2
8	7.00	7.9	13	4.4	4.7	7	6.2	6.2	0.1	5.5	2.7	2.8
9	10.3	11.6	13	5.4	6.2	15	44.6	35.2	21	11.5	5.4	6.1
10	4.8	5.5	15	2.7	3.2	19	16.5	10.6	36	8.4	4.0	4.4
11	3.9	6.0	54	2.0	3.3	65	6.1	4.0	34	4.4	3.9	0.5
12	7.6	7.9	39	4.6	4.8	4	40.6	40.1	1	3.6	2.1	1.5
p-value		p = 0.0001			P = 0.0003				P = 0.002			

**Figure 2:**
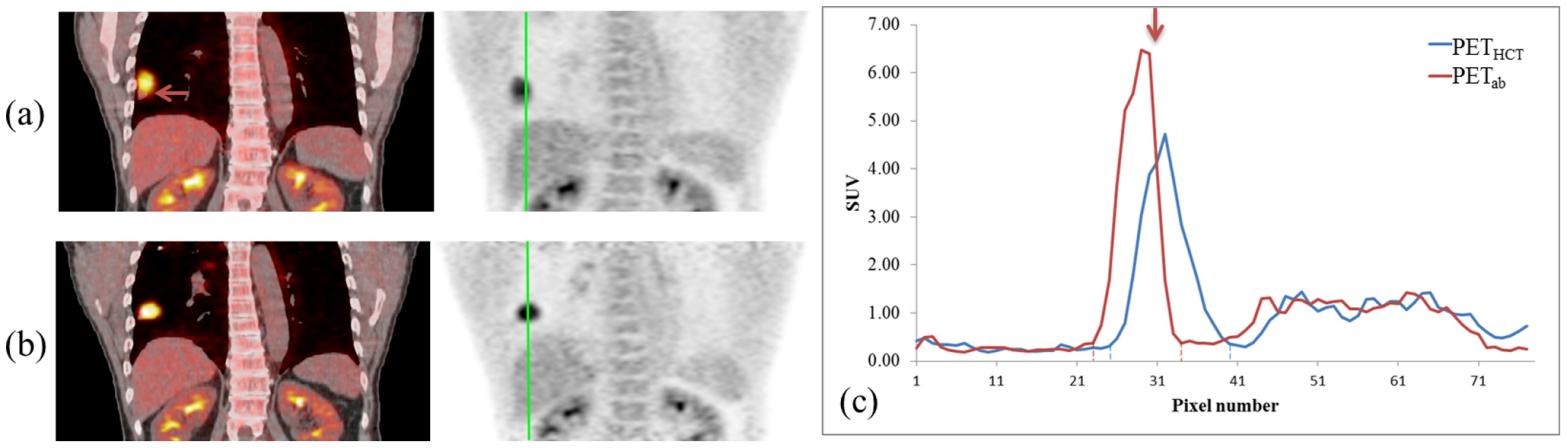
Coronal images of the (a) PET_FB_/CT fusion image (left); PET_FB_ (right) and (b) PET_ab_/CT_ab_ fusion image (left); and PET_ab_ (right) image for the selected patient, Patient 4. Misalignment around the tumor was observed in the PET_FB_/CT fusion images (red arrow). (c) Vertical image profiles are drawn across the tumor in the PET_FB_ and PET_ab_ images.

In Figure 2, the coronal views of the PET_FB_/CT and PET_ab_/CT_ab_ fusion images show the tumor in the right lower lobe for a selected patient, Patient 4, who was used as a representative example. Misalignment around the tumor (red arrow) was observed in the PET_FB_/CT fusion images and the misalignment was substantially improved in the PET_ab_/CT_ab_ image, as shown in Fig. 2(a) and Fig. 2(b), respectively. The values for PET_ab_ were greater than those for PET_FB_ by 8% and 13%. In addition, the vertical profiles drawn in Fig. 2(c) demonstrate that the full width at half maximum was smaller for the tumor shown in the PET_ab_ image, indicating less blurring around the edges of the tumor in the image, and a greater SUV_max_ was also easily observed in the PET_ab_ image, thereby enabling more accurate and precise tumor detection.

## Discussion

An abdominal compression device can be used to reduce lung tumor motion [18]. The efficiency of abdominal compression for reducing lung tumor motion depends on the tumor location within the lung. The significant effects of abdominal compression was assessed by Bouilhol et al [16]. The present study further demonstrated that PET/CT imaging incorporating abdominal compression potentially improved reconstructed PET image quality and produces increased SUVs of the tumors and reduced the respiratory artifacts containing spatial match in the PET and CT fusion images. Several concerns that may arise are that the increased SUV in the abdominal-compression images was caused by abdominal compression, or that in reality, the SUV will increase in active tumors with time postinjection because abdominal-compression PET acquisition was performed after conducting free-breathing PET acquisition on all of the patients. However, the results of this study revealed that the mean PD of SUV_max_ was 18%, which is too high to achieve time postinjection on the tumor within less than 5 minutes. In addition, the additional preparation time required to set up the abdominal compression device was typically less than 5 minutes in our clinical practice. Therefore, using the abdominal compression device for thoracic PET/CT acquisition is feasible for routine clinical use. There are two concerns regarding the use of abdominal compression: First, imaging combined with abdominal compression may cause discomfort and possible anxiety for some patients and is also unusable for obese patients. Second, abdominal compression might be a potential source of increased tumor motion variability, leading to inconsistencies in tumor delineation during simulation CT for radiation treatment planning [19]. To solve this problem, concatenating the deformable image registration to the abdominal compression is a possible option for linking simulation CT and CT_ab_ for delineating tumors [20].

Several studies have reported that a decrease in SUV in 3D PET scans is caused by the amount of displacement that occurs and the pattern of respiration motion. The 4D PET scan can be used to reduce the decrease in SUV induced by respiratory motion [6], [21]–[22]. This study demonstrated that PET imaging combined with abdominal compression device can also improve the SUV. Increases in both the SUV_max_ and SUV_mean_ for PET_ab_ compared with those for PET_FB_ were observed in this study. Tumors closer to the diaphragm clearly moved with a large amplitude in the superior-inferior direction; therefore, large SUV_max_ differences between 4D PET and 3D PET scans exist and have been reported in numerous studies. In this study, patient with tumors located in the middle to lower lobes of the lung were recruited and the SUV_max_ was successfully improved by approximately 7%–54%.

**Figure 3:**
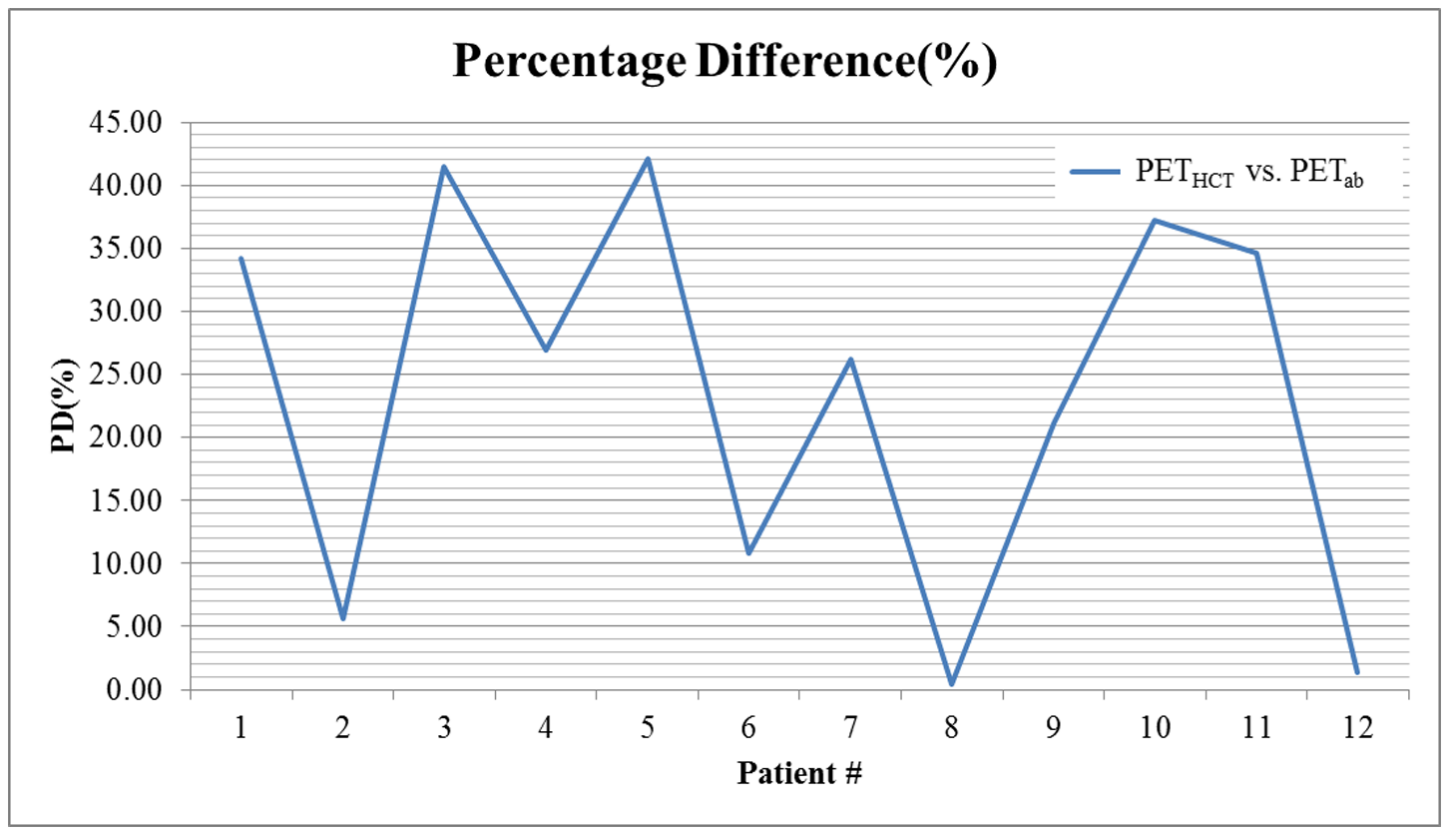
Percentage difference (PD %) in tumor volume derived from PET images of the patients.

The movement of the structures in the thorax is highly correlated to the diaphragm motion that occurs during respiration [23]. This movement typically causes a larger tumor volume size to appear in PET images, compared with the actual size of the tumor, leading to PET/CT misalignment [14]. The motion is even more complex when the lesions are attached to the rigid structure of the thorax, (eg, the pleura near the ribcage (Patients 3 and 10) and the diaphragm (Patient 5)). In this study, we observed significant differences in the quantification results, which indicated that the lesions attached to the rigid structure of the thorax demonstrated large volume changes (Fig. 3) between the images obtained with and without the use of abdominal compression. However, the effects of using abdominal compression on the lesion size, location, uptake ratio, and movement pattern are being further investigated in our current study.

## Conclusion

We provided the preliminary results regarding the differences in tumor motion caused by respiration in 12 lung cancer patients imaged using an abdominal compression device, compared with the images obtained using the conventional approach. The results demonstrated that the reduction in overall PET image quality resulted from respiratory motion and the mismatch between PET and CT caused by using CT for AC in PET to incorporate the abdominal compression device in PET/CT imagining.

## References

[1] Nehmeh SA, Erdi YE, Pan T, Pevsner A, Rosenzweig KE, et al (2004) Four-dimensional (4D) PET/CT imaging of the thorax. Med Phys 31(12): 3179–86. 15651600 10.1118/1.1809778

[2] Nehmeh SA, Erdi YE, Ling CC, Rosenzweig KE, Schoder H, et al (2002) Effect of respiratory gating on quantifying PET images of lung cancer. J Nucl Med 43(7): 876–81. 12097456

[3] Erdi YE, Nehmeh SA, Pan T, Pevsner A, Rosenzweig KE, et al (2004) The CT motion quantitation of lung lesions and its impact on PET-measured SUVs. J Nucl Med 45(8): 1287–92. 15299050

[4] Liu C, Pierce LA, Alessio AM, Kinahan PE (2009) The impact of respiratory motion on tumor quantification and delineation in static PET/CT imaging. Phys Med Biol 54(24): 7345–62. 19926910 10.1088/0031-9155/54/24/007 PMC2895622

[5] Huang TC, Wang YC (2013) Deformation effect on SUVmax changes in thoracic tumors using 4-D PET/CT scan. PLoS One 8(3): e58886. 23516568 10.1371/journal.pone.0058886 PMC3597593

[6] Nagamachi S, Wakamatsu H, Kiyohara S, Fujita S, Futami S, et al (2010) The reproducibility of deep-inspiration breath-hold 18F-FDG PET/CT technique in diagnosing various cancers affected by respiratory motion. Ann Nucl Med 24(3): 171–8. 20221810 10.1007/s12149-010-0352-3

[7] Boucher L, Rodrigue S, Lecomte R, Bénard F (2004) Respiratory gating for 3-dimensional PET of the thorax: feasibility and initial results. J Nucl Med 45(2): 214–9. 14960638

[8] Hamill JJ, Bosmans G, Dekker A (2008) Respiratory-gated CT as a tool for the simulation of breathing artifacts in PET and PET/CT. Med Phys 35(2): 576–85. 18383679 10.1118/1.2829875

[9] Wang YC, Tseng HL, Lin YH, Kao CH, Huang WC, et al (2013) Improvement of internal tumor volumes of non-small cell lung cancer patients for radiation treatment planning using interpolated average CT in PET/CT. PLoS One 8(5): e64665. 23696903 10.1371/journal.pone.0064665 PMC3655997

[10] Nehmeh SA, Erdi YE, Meirelles GS, Squire O, Larson SM, et al (2007) Deep-inspiration breath-hold PET/CT of the thorax. J Nucl Med 48(1): 22–6. 17204695

[11] Pan T, Mawlawi O, Nehmeh SA, Erdi YE, Luo D, et al (2005) Attenuation correction of PET images with respiration-averaged CT images in PET/CT. J Nucl Med 46(9): 1481–7. 16157531

[12] Huang TC, Mok GS, Wang SJ, Wu TH, Zhang G (2011) Attenuation correction of PET images with interpolated average CT for thoracic tumors. Phys Med Biol 56(8): 2559–67. 21444973 10.1088/0031-9155/56/8/014

[13] Mok GS, Sun T, Huang TC, Vai MI (2013) Interpolated average CT for attenuation correction in PET–a simulation study. IEEE Trans Biomed Eng 60(7): 1927–34. 23392338 10.1109/TBME.2013.2245132

[14] Sun T, Wu TH, Wang SJ, Yang BH, Wu NY, et al (2013) Low dose interpolated average CT for thoracic PET/CT attenuation correction using an active breathing controller. Med Phys 40(10): 102507. 24089928 10.1118/1.4820976

[15] Heinzerling JH, Anderson JF, Papiez L, Boike T, Chien S, et al (2008) Four-dimensional computed tomography scan analysis of tumor and organ motion at varying levels of abdominal compression during stereotactic treatment of lung and liver. Int J Radiat Oncol Biol Phys 70(5): 1571–8. 18374231 10.1016/j.ijrobp.2007.12.023

[16] Bouilhol G, Ayadi M, Rit S, Thengumpallil S, Schaerer J, et al (2013) Is abdominal compression useful in lung stereotactic body radiation therapy? A 4DCT and dosimetric lobe-dependent study. Phys Med 29(4): 333–40. 22617761 10.1016/j.ejmp.2012.04.006

[17] Bradley J, Thorstad WL, Mutic S, Miller TR, Dehdashti F, et al (2004) Impact of FDG-PET on radiation therapy volume delineation in non-small-cell lung cancer. Int J Radiat Oncol Biol Phys 59(1): 78–86. 15093902 10.1016/j.ijrobp.2003.10.044

[18] Keall PJ, Mageras GS, Balter JM, Emery RS, Forster KM, et al (2006) The management of respiratory motion in radiation oncology report of AAPM Task Group 76. Med Phys 33(10): 3874–900. 17089851 10.1118/1.2349696

[19] Bissonnette JP, Franks KN, Purdie TG, Moseley DJ, Sonke JJ, et al (2009) Quantifying interfraction and intrafraction tumor motion in lung stereotactic body radiotherapy using respiration-correlated cone beam computed tomography. Int J Radiat Oncol Biol Phys 75(3): 688–95. 19395200 10.1016/j.ijrobp.2008.11.066

[20] Guerrero T, Zhang G, Huang TC, Lin KP (2004) Intrathoracic tumour motion estimation from CT imaging using the 3D optical flow method. Phys Med Biol 49(17): 4147–61. 15470929 10.1088/0031-9155/49/17/022

[21] Aristophanous M, Berbeco RI, Killoran JH, Yap JT, Sher DJ, et al (2010) Clinical utility of 4D FDG-PET/CT scans in radiation treatment planning. Int J Radiat Oncol Biol Phys 82(1): e99–105. 10.1016/j.ijrobp.2010.12.060 21377285

[22] Callahan J, Binns D, Dunn L, Kron T (2011) Motion effects on SUV and lesion volume in 3D and 4D PET scanning. Australas Phys Eng Sci Med 34(4): 489–95. 22081269 10.1007/s13246-011-0109-x

[23] Cerviño LI, Chao AK, Sandhu A, Jiang SB (2009) The diaphragm as an anatomic surrogate for lung tumor motion. Phys Med Biol 54(11): 3529–41. 19443952 10.1088/0031-9155/54/11/017

